# Comparing the Impact of COVID-19 on Nurses’ Turnover Intentions before and during the Pandemic in Qatar

**DOI:** 10.3390/jpm11060456

**Published:** 2021-05-24

**Authors:** Abdulqadir J. Nashwan, Ahmad A. Abujaber, Ralph C. Villar, Ananth Nazarene, Mahmood M. Al-Jabry, Evangelos C. Fradelos

**Affiliations:** 1 Department of Nursing, Hazm Mebaireek General Hospital (HMGH), Hamad Medical Corporation (HMC), Doha 3050, Qatar; AABUJABER@hamad.qa (A.A.A.); RVillar@hamad.qa (R.C.V.); MAlJabry@hamad.qa (M.M.A.-J.); 2 Faculty of Nursing, University of Calgary in Qatar (UCQ), Doha P.O. Box 23133, Qatar; 3 Department of Nursing, Mental Health Services (MHS), Hamad Medical Corporation (HMC), Doha 3050, Qatar; ANazarene@hamad.qa; 4 Nursing Department, University of Thessaly, 41500 Larissa, Greece; evagelosfradelos@hotmail.com

**Keywords:** COVID-19, turnover intentions, intent to leave, TIS-6, nurses, Qatar

## Abstract

Background: Although several studies examining nurses’ turnover intentions have been conducted, few studies have been conducted to explore how COVID-19 contributes to nurses’ turnover intentions. This study aims to compare nurses’ turnover (TO) intentions before and during COVID-19. Methods: The cross-sectional study was conducted using the Turnover Intention Scale (TIS-6) and a convenience sample of participants from the largest healthcare provider in Qatar between August and September 2020. Results: A total of 512 nurses were included in the final analysis. The majority were between 31 and 40 years of age (61.5%), 67.6% were females, 76.4% were married, 79.7% had a BSN, 43% had less than 5 years of experience, and 60.4% had worked in COVID-19 designated facilities. The turnover intentions were higher compared with before COVID-19 (*p* < 0.01). Conclusion: Nurses in Qatar have higher TO intentions during COVID-19. The participants’ characteristics and stress levels are playing a major role in nurses’ decision to leave during COVID-19. Understanding the factors that contribute to turnover intentions is crucial for workforce planning, especially during pandemics.

## 1. Background

The nursing profession is considered to be a stressful and emotionally demanding profession; nurses in their everyday practice are dealing with various stressors, such as critical situations, workload, grief, death [1], nurses–physician collaboration and conflicts and uncertainty about the effectiveness of the provided care [2]. Moreover, the COVID-19 pandemic exposes nurses to additional stressors. According to a recent study that examined stressors in the nursing profession during the pandemic, in which 455 nurses participated, nurses nowadays have to deal with new sources of stress, such as personal protective equipment/supplies, exposure to sars-cov2 and the possibility of infection [3]. The constant exposure to stress many impact nurses’ personal and professional lives. Nurses that are exposed to various stressors are not satisfied with their profession or the provision of low-quality care [4], are experiencing secondary traumatic stress disorder and occupational burnout [5], and are adopting withdraw behaviors such as turnover and absenteeism [6].

Nurses’ turnover intentions have always been an international concern. Several research studies have been conducted to measure and have a better understanding of why nurses intend to leave their jobs. A systematic review undertaken by Halter and colleagues (2017) has shown that the most strongly supported determinants are at the individual level, which are stress and burnout, job dissatisfaction, and commitment [7]. These determinants became more significant as the demand for nurses is increasing in these crucial times brought by the coronavirus. The Coronavirus Disease 2019 (COVID-19) has increased the pressure on the healthcare system around the world. The virus continues to spread to more than 260 countries, infecting millions worldwide. During the COVID-19 pandemic, thousands of patients were admitted and the death toll was at an unspeakable rate. Nurses work for extended hours in an unsuitable working condition as hospitals reach maximum capacity. A large amount of studies proved that in pandemics such as COVID-19, nurses accumulates high stress and were at risk in developing mental health issues [8].

Nurses are working daily with the threat of getting infections. Unfortunately, there is no detailed report on the number of nurses who got infected with COVID-19. However, The International Council of Nurses (ICN) reported on 3 June 2020 that more than 600 nurses died from the 450,000 healthcare workers who acquired the virus [9]. Zhang and Small (2020) estimated that 1652 healthcare workers died fighting COVID-19 as of May 2020 through the capture and recapture method. The thought of being infected among nurses may increase their defensive behavior and decrease their willingness to work with infected patients [8,10,11,12,13,14,15]. Labrague and De Los Santos (2020) support that the fear of being infected with the coronavirus among nurses impacts their intent to leave the profession and organization. In previous pandemics (e.g., H1N1), the absenteeism rate among nurses was higher during outbreaks compared to normal situations [13,16,17].

Several research studies have been conducted to have a better understanding of the reasons behind the intention of nurses to leave their jobs. To date, few studies have been conducted to explore how COVID-19 contributes to nurses’ turnover intentions. This study aims is to explore and compare nurses’ turnover intentions in Qatar during and before the COVID-19 pandemic.

## 2. Methods

This cross-sectional, survey-based descriptive study was conducted in Hamad Medical Corporation (HMC) from August 2020 to September 2020. After receiving approval from the International Review Board (IRB), a survey link was disseminated to approximately 12,000 nurses through the corporate’s official email. Participants were asked to declare whether they were assigned or deployed to a COVID-19 facility or not. We allocated one month for data collection and several reminders were sent to improve the response rate.

### 2.1. Data Collection

The first part of the survey included the participants’ characteristics: gender, age, marital status, years of experience, education level, whether they are working with COVID-19 patients or not, role, area of expertise (the area or department of work), and deployment during COVID-19 (duration of assignment in a COVID-19 facility). To measure the difference of stress levels between the stress before COVID-19 and during the COVID-19, the nurses were asked to recall their stress levels before COVID-19 and compare it to their day-to-day stress during the COVID-19 pandemic. A questionnaire was added asking the participant to categorize their stress levels into a five-point Likert scale (no stress at all, mild, moderate, much, and extreme stress).

The widely used Turnover Intention Scale (TIS-6) is a 6-item self-administered questionnaire that was adapted from Roodt’s (2004) original 15-item scale. A five-point Likert scale was used to rate the responses. The midpoint of the scale is 18. The scores below 18 indicate a desire to stay. If the scores are above 18, it indicates a desire to leave the organization. The minimum score a participant can get is 6 and the maximum is 30. There are no reverse scores in the TIS-6. The study undertaken by Bothma and Roodt (2013) confirmed that the TIS-6 is a reliable and valid tool to construct and predict actual turnover intention and behavior (α = 0.80) [18]. In order to compare the participant’s TIS-6 score before COVID-19 and during COVID-19, the nurses were asked to answer two sets of TIS-6. The nurses were asked to recall their feelings and emotions before COVID-19 when answering the first set of TIS-6 and during COVID-19 when answering the second set of TIS-6. Please see Supplementary Tables S1 and S2.

### 2.2. Ethical Approval

This research project has been reviewed and approved by the IRB at the Medical Research Center (MRC) in Hamad Medical Corporation (MRC-01-20-609), and the study has been conducted in full conformance with the principles of the “Declaration of Helsinki” for Good Clinical Practice (GCP). The invitation to participate was sent to all nurses via the HMC official corporate email, along with a cover letter explaining the study’s purpose and expectations. Informed consent was obtained from all study participants in the survey. After data collection, the data were coded and double-checked by the principal investigator; the data were stored in a protected computer following Hamad Medical Corporation (HMC)’s policies and guidelines.

## 3. Statistical Analysis

The Statistical Package for the Social Sciences (SPSS), version 25.0, was used to analyze the data (the statistical significance level at (0.05)). Descriptive statistics were used to analyze the distributions of responses on the questionnaire items and subscales. Inferential statistics were focused on finding the correlation between different variables. The participants’ scores on the different variables were compared with the independent samples *t*-test, one-way analysis of variance (ANOVA), Shapiro–Wilk’s test, and the Wilcoxon signed-ranks test as appropriate. Multivariable linear regression analysis using all the demographic variables as independent variables and turnover overall score as the outcome/dependent variable was conducted to identify factors associated with turnover, and predict multiple logistic regression analysis was performed to identify significant predictors of turnover intentions. Unstandardized regression coefficients (β) at 95% confidence intervals (CIs) were used to quantify the associations between variables.

## 4. Results

The data were collected from a total of 512 nurses (4.3% of the total number of nurses working at HMC). The sample was calculated based on the number of nurses working in HMC and with a CI of 95% (50% the response distribution and 5% margin of error), the estimated sample size was 373. We increased it to 500+ to overcome any incomplete surveys. *Participants’ demographics.* The majority were between 31 and 40 years of age (61.5%). Among the study participants, 67.6% were females, 76.4% were married, 79.7% were BSN graduated, 43% of them were with less than 5 years of experience in the field, and 60.4% were assigned to a COVID-19 designated facility during the pandemic. A total of 40% of the participants were working at the bedside, 40% were not deployed during the crisis, and 47% were deployed in a COVID-19 facility for less than 6 months (Table 1).

### Stress Levels during and before COVID-19

There was a significant difference between turnover intentions before and during COVID-19; the turnover intentions increased during COVID-19 (Table 2). The below table highlights the eminent difference, as the subjects with no stress and mild stress were decreased from 8.8% and 49% to 2.1% and 15.8%, respectively, during COVID-19. Other participants with much and extreme stress were increased from 12.7% and 3.9% to 33.2% and 17.6%, respectively. Thus, the stress among staff was significantly increased compared to the level of stress before COVID-19.

The Shapiro–Wilk’s test results were (0.00, 0.00) for turnover intentions both before and during COVID-19, respectively (*p* > 0.05), were approximately normally distributed with a skewness of 0.705 (SE = 0.108) and a kurtosis of 0.216 (SE = 0.215) for turnover intentions before COVID-19, and turnover intentions during COVID-19 show a skewness of 0.433 (SE = 0.108) and a kurtosis of 0.645 (SE = 0.215) for turnover during COVID-19. The values for asymmetry and kurtosis between −2 and +2 were considered acceptable to prove normal univariate distribution [19].

Table 3 shows the result derived from the Wilcoxon signed-ranks test, which indicates that turnover intentions of nurses during COVID-19 were statistically significantly higher than turnover before COVID-19 (Z = −11.058) (*p* < 0.01) and further highlights the association between demographic variables and turnover intentions among nurses. The Mann–Whitney test has indicated no significant association between gender difference and turnover intentions before or during COVID-19.

There was a statistically significant difference between the age groups of the study participants on turnover intentions before (Kruskal–Wallis H = 8.878), with a high mean rank of 280.48 for the age group of 51 years and with a low mean rank of 215.72 for the age group of between 21 and 30 years of age with *p* = 0.031. Moreover, most of the age groups, except the 21–30 years of age group, have more turnover intentions before COVID-19 with a high mean rank of 265.57 for the age group 31–40 years and with the low mean rank of 224.53 for the age group of between 21 and 30 years of age with *p* = 0.125 during COVID-19.

The participants with more than 5 years of experience merely have the same turnover intentions before COVID-19, while the participants between 31 and 50 years of age merely have the same turnover intentions during COVID-19. The distribution of turnover intentions before/during is the same across categories of age with the significance level of 0.031.

Meanwhile, the authors also found that there was a statistically significant difference among the different marital groups of the study participants on turnover intentions before (Kruskal–Wallis H = 5.224) and during (Kruskal–Wallis H = 9.885), with a high mean rank of 283.98 among the single (unmarried) group and a low mean rank of 248.28 among the married group with *p* = 0.073 (before), and a high mean rank of 294.41 among the single (unmarried) group and a low mean rank of 225.00 among the other categories of the marital status group with *p* = 0.007 (during). The participants with a single state have more than the other participants with married and other marital statuses of turnover intentions during COVID-19. The distribution of turnover intentions during COVID-19 is the same across categories of marital status with the significance level of 0.007.

Furthermore, this study reveals that there was a statistically significant difference among the groups with varying years of experience among the study participants on turnover intentions before (Kruskal–Wallis H = 14.983) and during (Kruskal–Wallis H = 5.744), with a high mean rank of 280.96 for the participants with 5–10 years of experience, and participants with less than 5 years of experience have a low mean mark of 227.52 with *p* = 0.001 before. From the above tables, the results summarize that there was a statistically significant difference among the groups with different years of experience of the study participants on turnover intentions during COVID-19 (Kruskal–Wallis H = 7.587), with a high mean rank of 282.19 for the participants with 5–10 years of experience, and participants with less than 5 years of experience have a low mean mark of 240.48 with *p* = 0.023.

The findings conclude that the participants with 5–10 years of experience have more turnover intentions during COVID-19 than the other groups who merely possess the same turnover intentions during COVID-19.

The results show a statistical significance among the study participants in terms of stress before COVID (Kruskal–Wallis H = 47.952), with a mean of 333.63 with extreme stress and a mean of 186.60 with no stress with *p* = 0.000. The most significant number is for participants with mild stress before COVID-19, then participants who are with either moderate, much, or extreme stress, with very few participants having no stress. The above tables show that there is statistical significance.

As far as the education level is concerned, it is evident from this study’s findings that there was a statistically non-significant difference among the groups with different educational qualifications of the study participants on turnover intentions before (Kruskal–Wallis H = 0.245), a high mean rank of 265.14 for the participants with nursing graduation and diploma graduate participants with 253.49 low mean ranks with *p* = 0.885. The participants with any educational qualification, except graduates, merely have the same turnover intentions before COVID-19, while graduates posed more turnover intentions before COVID-19. Meanwhile, there is a statistically significant difference among the groups with different educational qualifications of the study participants on turnover intentions during (Kruskal–Wallis H= 0.827), a high mean rank of 270.44 for the participants with nursing graduation and diploma graduate participants with 244.79 low mean ranks with *p* = 0.661. These tables help to conclude that the participants with any educational qualification, except just graduates, merely have the same turnover intentions during COVID-19, while graduates posed more turnover intentions during COVID-19.

For the clinical expertise, there is a statistically significant difference among the study participants with different expertise of the on turnover intentions before (Kruskal–Wallis H = 4.509), with a high mean rank of 276.62 among the participants with critical care expertise and participants with med/surgical expertise with 246.31 low mean rank with *p* = 0.342. The participants with critical care and emergency expertise have more turnover intentions before COVID-19 than participants with other expertise. Furthermore, these studies found that there is a statistically significant difference among the study participants with different expertise on turnover intentions during COVID-19 (Kruskal–Wallis H = 3.275), with a high mean rank of 271.17 among the participants with critical care expertise and participants with other than the listed expertise with 241.55 low mean rank with *p* = 0.513. These tables help to establish the statement that the participants with critical care expertise have more turnover intentions during COVID-19 than participants with other expertise, while the participants with medical/surgical and emergency, pediatric, and other expertise merely have the same turnover intentions during COVID-19.

All participants, irrespective of whether they were deployed in the COVID-19 facilities or not, have turnover intentions during COVID-19 (*p* = 0.047). Understandably, there is a statistically significant difference among the study participants with a different area of deployment on turnover intentions during COVID-19 (Kruskal–Wallis H = 9.613), with a high mean rank of 297.78 among the participants with critical care deployment and participants with other than the listed main areas of deployment with 242.90 low mean rank with *p* = 0.047. Further, the hypotheses test summary by SPSS with a *p*-value of 0.047 states that the distribution of turnover intentions is the same across categories of deployment.

The data from this study lead to the conclusion that there is a statistically significant difference among the study participants with varying durations of deployment on turnover intentions during COVID-19 (Kruskal–Wallis H = 1.833), with a high mean rank of 160.47 among the participants with 3–6 months of deployment and participants with less than 2 months of deployment with 145.68 low mean rank with *p* = 0.047. This means that turnover intentions during COVID-19 were high among the participants with 3–6 months of deployment in a COVID-19 facility; after 6 months, their turnover intentions during COVID-19 were less, reaching the same as those of the participants deployed for less than 2 months.

## 5. Discussion

This study was trying to measure the impact of COVID-19 on nurses’ turnover intentions in Qatar. To the researcher’s knowledge, this is the first study to compare the turnover intentions of nurses before COVID-19 and during COVID-19. The results show that nurses in Qatar have significantly higher turnover intentions during COVID-19 compared to before COVID-19 (Z = −11.058) (*p* < 0.01). This result is in agreement with various studies worldwide [20,21,22]. The COVID-19 pandemic has added an extra burden to the already exhausted nursing personnel; according to a recent systematic review and metanalyses, 27% of nurses are experiencing high levels of anxiety during the COVID-19 pandemic [23]. In a cross-sectional study of 261 frontline nurses in the Philippines, nurses were experiencing high levels of fear of COVID-19 and extend psychological distress, which may be associated with the increased percentage of nurses’ turnover intention [24].

It is true in some studies that nurses have a high turnover intention because of the psychological response to fear of COVID-19 [24,25,26]. In addition, this study shows that stress is significantly higher among nurses during COVID-19 compared to before COVID-19. This is consistent with previous studies where nurses accumulate higher stress during pandemics and is a strong determinant for nurses to leave their jobs [8,27]. There are various factors associated with low rates of work engagement and increased rates of turnover intention. A cross-sectional study in Sweden, in which 807 registered nurses participated, revealed that work–family conflict can be a strong predictor in nurses’ turnover intention. Thus, managers should promote a positive work climate and a balance between nurses’ work and family life in order to keep nurses in their profession [28].

It is important to discover other factors associated with the nurses’ stress and turnover intention during COVID-19 in order to mitigate and retain nurses during pandemics.

Furthermore, in this study, nurses above 30 years old have higher turnover intentions during COVID-19, while nurses aged 21–30 years old have significantly lower turnover intentions than before COVID-19.

Multiple studies support that age significantly influences turnover intentions [24,29,30]. In addition, nurses working in Qatar for 5–10 years have higher turnover intentions than nurses working for less than 5 years before and during COVID-19. According to a recent European study in which a cohort of 753 nurses participated, experienced and older nurses were less likely to exhibit turnover behaviors [31]. Furthermore, similar findings are reported in various studies, in which, in normal circumstances, the young, single, and new nurses had a higher tendency to leave their jobs [32,33]. A probable explanation would rest on the nurse’s perception of stability, work life–family balance, and health concerns during COVID-19. The findings were alarming for hospital managers since the senior and more experienced nurses were considering leaving their jobs.

Another finding is that the length of deployment to a COVID-19 facility also affects a nurse’s turnover intentions. This study shows that nurses who worked in a COVID-19 facility for more than three months have a significantly higher turnover intention than those who did not work in a COVID-19 facility. The study suggests that hospitals should consider decreasing the deployment period of nurses, especially for nurses deployed in critical care. The study also showed that critical care nurses have higher turnover intention scores compared to other participants who were assigned elsewhere during COVID-19. Different studies have shown that even before the COVID-19 pandemic nurses in the critical care units have higher stress and are more dissatisfied because of their continuous exposure to traumatic and stressful work experience, resulting in higher turnover intentions [34,35]. In the state of Qatar, critical care units were expanded to admit more critical patients; nurses without or minimal critical care experience have undergone crash courses or trainings in order to be safely and efficiently deployed in ICUs to fill the workforce [36]. Critical care nurses witness a lot of patient suffering, which caused a lot of emotional distress and grief during the COVID-19 pandemic [8].

It is also interesting to note that in the study of Nashwan et. al. (2021) the nurses who were more knowledgeable and less exposed were willing to take care of patients infected with COVID-19 in Qatar [37]. Similar results were also reported by Lord et al. (2021), in an Australian sample of ICU nurses, where good communication, the amount of knowledge and preparedness were significant predictors of willingness to care for COVID-19 patients [38]. A possible explanation is that although nurses find it an obligation to work during COVID-19, the stress and the nurses’ characteristics during COVID-19 were important factors in their decision to leave their work [8,10,24].

## 6. Limitations

Despite its adequate methodology and findings, the present study has certain limitations. There are some major limitations in this study that could be discussed in future research—first, the electronic form of administration can raise some concern in terms of participation. This means that only those that had an active email account and access to it could participate, which raises a possibility of selection bias. Although the invitation was sent to about 12,000 nurses working in HMC, the response rate was only 4.3%. The non-respondents were either too stressed to complete the survey or not interested in participating. Second, the survey was carried out months after the peak of COVID-19; the stress level and TIS-6 were asked as recall questions with a possible recall bias. Finally, turnover intention before the pandemic was measured later during the pandemic, and time as well as circumstance could alter their responses.

## 7. Conclusions

The COVID-19 pandemic exerted a huge influence on nurses worldwide. The findings of this study have important implications for nursing leaders, as they highlight the difference between nurses’ turnover intentions before and during the COVID-19 pandemic. Nursing leaders need to exhaust all possible measures and resources to support frontline nurses and address their physical, mental, and social needs. Supporting nurses through developing a safe and healthy working environment is essential. Evidence-based strategies should be followed by nursing leaders to retain and support nurses all the time, especially during pandemics. Consistent with prior evidence, our study findings suggest that nurses in Qatar have higher turnover intentions during compared to before COVID-19. Participants’ characteristics and stress levels are playing a major role in nurses’ decision to leave during COVID-19. Understanding the factors that contribute to the turnover intentions is crucial for workforce planning, especially during pandemics. Addressing the factors associated with turnover intentions will positively be reflected in nurses’ well-being and turnover intentions.

## Figures and Tables

**Table 1:** Demographics of the participants.

Characteristics	Categories	N	Percentage (%)
**Gender**	Male	166	32.4
	Female	346	67.6
**Marital status**	Single	115	22.5
	Married	391	76.4
	Other	6	1.2
**Education**	Diploma	43	8.4
	BSN	408	79.7
	Graduate Studies	31	11.9
**Assigned to a COVID-19 facility**	Yes	309	60.4
	No	203	39.6
**Role during crisis**	Bedside nurse	205	40
	Charge Nurse, Coordinator	307	60
**Original unit of assignment**	Med/surg	138	27
	Critical Care	93	18.2
	Emergency	78	15.2
	Pediatrics	48	9.4
	Other	155	30.3
**Area of assignment in a COVID-19 facility**	Not Deployed	205	40
	Critical Care	86	16.8
	Emergency	49	9.6
	Quarantine Facilities	40	7.8
	Other	132	25.8
		Mean	SD
**Age**		36.54	7.42
**Years of experience as a nurse**		6.54	4.46
**Deployment duration in a COVID-19 facility (n = 307)**		4.36	2.41
		**Frequency**	**Percentage**
**Stress level during COVID-19**	No stress	11	2.1
	Mild	81	15.8
	Moderate	160	31.3
	Much	170	33.2
	Extreme	90	17.6
**Stress level before COVID-19**	No stress	45	8.8
	Mild	251	49
	Moderate	131	25.6
	Much	5	12.7
	Extreme	20	3.9

**Table 2:** Stress levels before and during COVID-19.

	Statistic	df	Sig.	Mean	95% CI
TO before COVID-19	0.105	512	0.000	13.24	12.83–13.66
TO during COVID-19	0.089	512	0.000	15.54	15.03–16.04

**Table 3:** Comparison of TO intentions before and during COVID-19.

	Turnover Intention Sig. (2-Tailed)	
	Before COVID-19	During COVID-19
Gender	0.259	0.859
Age	0.031 *	0.125
Marital status	0.073 *	0.007 *
Years of experience	0.001 *	0.023 *
Education	0.885	0.661
The original field of expertise	0.342	0.513
Working in a COVID-19 designated facility	-	0.648
Role during the pandemic	-	0.136
Deployment	-	0.047 *
Deployment duration	-	0.400
Stress level	0.000 *	0.000 *

* statistically significant.

## Data Availability

All data generated during this study are included in this published article.

## Supplementary Materials

The following are available online at https://www.mdpi.com/article/10.3390/jpm11060456/s1, Table S1: Turnover Survey Questionnaire, Table S2: DURING THE PAST 9 MONTHS (during COVID-19)
