# Supplementary material for: Comparing the Impact of COVID-19 on Nurses’ Turnover Intentions before and during the Pandemic in Qatar
Source: J Pers Med. 2021 May 24;11(6):456. doi: 10.3390/jpm11060456 (PMC8225037; doi:10.3390/jpm11060456)
Supplement: Supplementary file 1 [file jpm-11-00456-s001.zip › jpm-1193248-supplementary.pdf]

Supplementary

Table S1. Turnover Survey Questionnaire

I. Demographics

1. Gender
   a. Male
   b. Female
2. Age
   a. 21-30
   b. 31-40
   c. 41-50
   d. 51-60
   e. >60
3. Marital status.
   a. Single
   b. Married
   c. Widowed
   d. Separated
4. Years of Experience in HMC
   a. Less than 1 year
   b. 1 year and more
5. Years of Experience in Nursing (total)
   a. Less than 5 years
   b. 5 to 10 years
   c. More than 10 years
6. Level of Education
   a. Diploma
   b. Bachelor's
   c. Masters
   d. PhD
7. Are you a registered nurse who's working during the COVID-19 pandemic?
   a. Yes
   b. No
8. Are you directly taking care of patients with COVID-19?
   a. Yes
   b. No
9. What best describes your role during the COVID-19 pandemic?
   a. Bedside nurse
   b. Charge Nurse/Nurse Manager/Coordinator
   c. Leadership or Administrative
   d. Education
   e. Others (please specify)
10. Please select your (original) field of expertise:

   a. Medical/Surgical
   b. Critical care
   c. Emergency
   d. Pediatrics
   e. Mental Health
   f. Others (Please specify):
11. Where have you been redeployed during the COVID-19 pandemic?
   a. Not redeployed
   b. Critical care
   c. Emergency
   d. Quarantine facilities
   e. Other (specify)
12. If you would rate your stress level during the COVID-19 pandemic, how would you rate it?

No stress at all	Mild Stress	Moderate Stress	Much Stress	Extreme Stress
1	2	3	4	5

13. If you would rate normal day to day stress (pre-COVID 19), how would you rate it?

No stress at all	Mild Stress	Moderate Stress	Much Stress	Extreme Stress
1	2	3	4	5

TURNOVER INTENTION SCALE (TIS)

Copyright © 2004, G. Roodt
The following section aims to ascertain the extent to which you intend to stay at the organization. Please read each question and indicate your response using the scale provided for each question:
Table S2 DURING THE PAST 9 MONTHS (during COVID-19)

1	How often have you considered leaving your job?<br>A. Before Covid-19<br>B. During Covid-19	Never	1-------2-------3-------4-------5	Always
2	How frequently do you scan the newspapers/internet in search of alternative job opportunities?	Never		All the time

			1-------2-------3-------4-------5	
3	How satisfying is your job in fulfilling your personal needs?	Very satisfying	1-------2-------3-------4-------5	Totally dissatisfying
4	How often are you frustrated when not given the opportunity at work to achieve your personal work-related goals?	Never	1-------2-------3-------4-------5	Always
5	How often are your personal values at work compromised?	Never	1-------2-------3-------4-------5	Always
6	How often do you dream about getting another job that will better suit your personal needs?	Never	1-------2-------3-------4-------5	Always
7	How likely are you to accept another job at the same compensation level should it be offered to you?	Highly unlikely	1-------2-------3-------4-------5	Highly likely
8	How often do you look forward to another day at work?	Always	1-------2-------3-------4-------5	Never
9	How often do you think about starting your own business?	Never	1-------2-------3-------4-------5	Always
10R	To what extent do responsibilities prevent you from quitting your job?	To no extent	1-------2-------3-------4-------5	To a very large extent

11R	To what extent do the benefits associated with your current job prevent you from quitting your job?	To no extent	1-------2-------3-------4-------5	To a very large extent
12	How frequently are you emotionally agitated when arriving home after work?	Never	1-------2-------3-------4-------5	All of the time
13	To what extent does your current job have a negative effect on your personal well-being?	To no extent	1-------2-------3-------4-------5	To a very large extent
14R	To what extent does the "fear of the unknown", prevent you from quitting?	To no extent	1-------2-------3-------4-------5	To a very large extent
